# Inhibition of RPA32 and Cytotoxic Effects of the Carnivorous Plant *Sarracenia purpurea* Root Extract in Non-Small-Cell Lung Cancer Cells

**DOI:** 10.3390/plants14101426

**Published:** 2025-05-09

**Authors:** Kuo-Ting Chang, Yu-Cheng Chen, Yi Lien, Yen-Hua Huang, Cheng-Yang Huang

**Affiliations:** 1 Division of Translational Medicine, Department of Research and Development, Taoyuan General Hospital, Ministry of Health and Welfare, Taoyuan 330, Taiwan; 2 Division of Pulmonary Medicine, Department of Internal Medicine, Taoyuan General Hospital, Ministry of Health and Welfare, Taoyuan 330, Taiwan; 3 Department of Biological Sciences, Purdue University, West Lafayette, IN 47907, USA; 4 Department of Biomedical Sciences, Chung Shan Medical University, Taichung 402, Taiwan; 5 Department of Medical Research, Chung Shan Medical University Hospital, Taichung 402, Taiwan

**Keywords:** *Sarracenia purpurea*, anticancer, RPA, ssDNA binding, AlphaFold, NSCLC, H1975, α-amyrin, ursolic acid, betulinaldehyde

## Abstract

The carnivorous plant *Sarracenia purpurea* has been traditionally used in various ethnobotanical applications, including treatments for type 2 diabetes and tuberculosis-like symptoms. This study investigates the cytotoxic effects of *S. purpurea* root extract (Sp-R) on human non-small-cell lung cancer (NSCLC) cell lines, including H1975, H838, and A549, focusing on its impact on cell survival, apoptosis, proliferation, and migration. Additionally, its ability to inhibit the single-stranded DNA-binding activity of human RPA32 (huRPA32), a key protein in DNA replication, was evaluated. Extracts from different plant parts (leaf, stem, and root) were prepared using various solvents (water, methanol, ethanol, and acetone) and screened for apoptosis-inducing potential using the chromatin condensation assay. Among these, the acetone-extracted root fraction (Sp-R-A) exhibited the most potent pro-apoptotic effects. The MTT assay demonstrated a dose-dependent cytotoxic effect on NSCLC cells, with IC_50_ values of 33.74 μg/mL for H1975, 60.79 μg/mL for H838, and 66.52 μg/mL for A549. Migration and clonogenic assays further revealed that Sp-R-A significantly inhibited cancer cell migration and colony formation in a dose-dependent manner. Moreover, Sp-R-A enhanced apoptosis when combined with the EGFR inhibitor afatinib, suggesting a potential synergistic effect. The electrophoretic mobility shift assay confirmed that Sp-R-A significantly inhibited the DNA-binding activity of huRPA32, with an IC_50_ of 13.6 μg/mL. AlphaFold structural prediction and molecular docking studies indicated that major bioactive compounds in *S. purpurea*, including α-amyrin, ursolic acid, and betulinaldehyde, strongly interact with the DNA-binding domain of huRPA32, potentially contributing to its inhibitory effect. Overall, these findings suggest that huRPA32 is a potential molecular target of Sp-R-A and the anticancer potential of *S. purpurea* root extract against NSCLC is highlighted, supporting further investigation into its therapeutic applications.

## 1. Introduction

Natural products, particularly plant-derived compounds and secondary metabolites have played a crucial role in drug discovery, offering structurally diverse bioactive molecules with potential therapeutic applications, including cancer treatment [1,2,3,4]. The carnivorous plant *Sarracenia purpurea* [5,6,7] is recognized as a medicinal plant in Canada [8] and has been traditionally used in ethnomedicine for treating ailments such as type 2 diabetes [9,10] and tuberculosis-like symptoms [11]. However, its cytotoxic effects against cancer cell lines remain largely unexplored and warrant experimental validation. *S. purpurea*, commonly known as the purple pitcher plant, northern pitcher plant, turtle socks, or side-saddle flower, belongs to the Sarraceniaceae family [12]. It is the only member of this genus adapted to cold temperate zones and is predominantly found in nutrient-poor environments such as sphagnum bogs, poor fens, seepage swamps, and pine savannas across the eastern and northern regions of the United States and Canada [13]. Carnivory in plants is a rare evolutionary adaptation that involves distinctive morphological modifications, primarily in leaf structures, to facilitate survival in nutrient-deficient habitats [14,15]. These modifications enable carnivorous plants to supplement their nutrient intake, particularly nitrogen and phosphorus, by capturing and digesting prey. Similarly to other members of the *Sarracenia* genus, *S. purpurea* relies on passive pitfall traps formed by its specialized pitcher-shaped leaves to capture insects and other small organisms [16]. However, its prey capture efficiency is relatively low, with less than 1% of visiting organisms being successfully trapped [17]. Examples of prey species commonly include flies, ants, spiders, moths, hornets, and other invertebrates [18,19]. Given the unique adaptations of *S. purpurea* for acquiring nutrients, it remains unknown whether its plant body exhibits cytotoxic activity or specificity toward cancer cells. Investigating the potential cytotoxic and bioactive properties of *S. purpurea* is, therefore, of significant interest, as it may harbor novel compounds with anticancer and other therapeutic applications.

Lung cancer remains a leading cause of cancer-related mortality worldwide, accounting for approximately 1.8 million deaths annually [20]. Non-small-cell lung cancer (NSCLC), which constitutes around 85% of all lung cancer cases, is conventionally treated with surgery, chemotherapy, radiation therapy, and targeted therapies [21,22]. However, single-agent treatments often drive cancer cells to activate alternative survival pathways, ultimately leading to drug resistance [23,24,25]. Notably, therapy resistance accounts for nearly 90% of cancer treatment failures and contributes to 80–90% of cancer-related deaths [26]. Therefore, the integration of multiple chemotherapeutic agents, including natural compounds, is a promising strategy for overcoming resistance and identifying alternative therapeutic approaches for lung cancer treatment [27,28,29]. Natural products have emerged as a valuable resource for drug discovery, with more than 30% of all U.S. Food and Drug Administration (FDA)-approved drugs being either natural products or their derivatives [30,31,32]. Given this potential, we investigated the cytotoxic effects of various *S. purpurea* extracts, prepared using different solvents, on NSCLC cell survival, migration, proliferation, and apoptosis in A549, H838, and H1975 cells. Additionally, the acetone-extracted root fraction (Sp-R-A) enhanced the cytotoxic effect when combined with the clinically approved anti-lung cancer drug afatinib, suggesting a potential synergistic interaction.

The identification of new molecular targets for NSCLC remains a critical area of investigation [33,34,35,36], particularly in light of the promising anticancer potential and cytotoxic effects of *S. purpurea* extracts. To date, only one protein involved in DNA metabolic processes such as replication, repair, recombination, and replication fork restart has been reported to be inhibited by *S. purpurea* extracts: the bacterial single-stranded DNA (ssDNA)-binding protein (SSB) SsbA [37,38,39]. SSB plays an essential role in bacterial DNA replication and cell proliferation by utilizing oligonucleotide/oligosaccharide-binding (OB) folds for ssDNA binding [40,41,42,43,44,45]. Notably, the eukaryotic counterpart of bacterial SSB is Replication Protein A (RPA), which serves analogous mechanistic functions in DNA replication and genome stability [46,47,48,49]. RPA, an OB-fold-containing heterotrimeric complex, consists of three subunits: RPA70, RPA32, and RPA14; each of these possess distinct functional roles. Among these, RPA32 is crucial for ssDNA binding and interacts with various genome maintenance proteins, including RAD52, TIPIN, XPA, UNG2, and SMARCAL1 [50,51]. The inhibition of the RPA function induces replication stress, suppresses tumor growth [52], and contributes to cell death following irradiation [53]. Furthermore, the downregulation of RPA32 foci formation has been implicated in the suppression of NSCLC progression [54]. Given that normal human cells do not require continuous DNA synthesis, an inhibitor specifically targeting RPA32 may selectively induce cytotoxicity in rapidly proliferating cancer cells that rely on sustained genome replication. Considering the structural and functional similarities between SsbA and RPA32, we investigated whether *S. purpurea* extracts could inhibit the ssDNA-binding activity of human RPA32 (huRPA32), which is a key protein in DNA replication. Our findings suggest that huRPA32 may serve as a potential molecular target in NSCLC therapy. Additionally, AlphaFold’s structural prediction and various molecular docking studies revealed that major bioactive compounds in *S. purpurea* [8,11,55], including α-amyrin, ursolic acid, and betulinaldehyde, strongly interact with the ssDNA-binding domain of huRPA32, potentially contributing to its inhibitory effect. These findings indicate that *S. purpurea* may serve as a natural source of RPA32 inhibitors and highlight its potential application as a complementary therapeutic strategy for NSCLC treatment.

## 2. Results

### 2.1. Apoptosis Screening of Sarracenia purpurea Extracts Against Lung Cancer Cells

**Figure 1:**
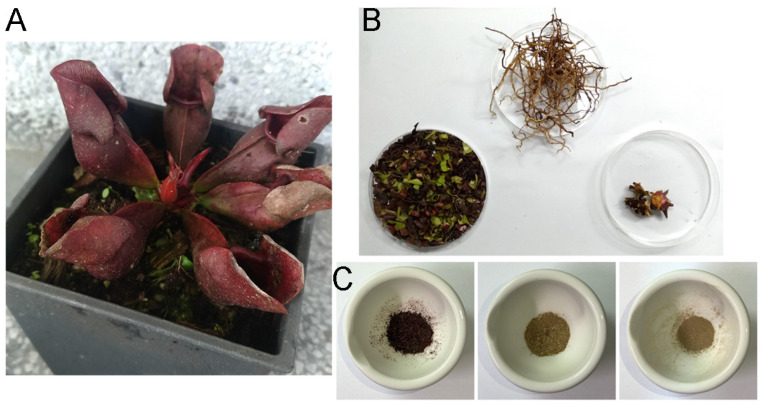
The preparation of *S. purpurea* extracts. (**A**) *S. purpurea*: a carnivorous pitcher plant characterized by cone-shaped leaves adapted for nutrient acquisition. (**B**) Different parts of *S. purpurea*, including the leaves (**left**), roots (**middle**), and stems (**right**), were collected, dried, cut into small pieces, and pulverized into fine powder. (**C**) Pulverized powder of *S. purpurea* leaves (**left**), roots (**middle**), and stems (**right**). Extraction was performed using polar protic solvents (methanol, ethanol, and distilled water) and a polar aprotic solvent (acetone) for subsequent analyses.

**Figure 2:**
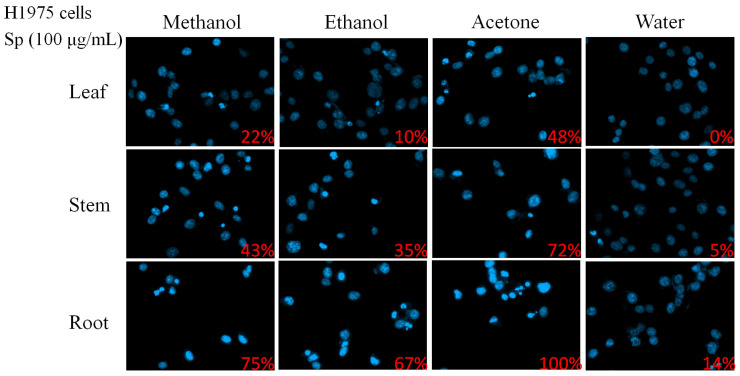
The cytotoxic effects of Sp extracts on apoptosis induction in H1975 cells. The chromatin condensation assay using Hoechst staining was performed to assess DNA fragmentation, which is a key hallmark of apoptosis. H1975 cells were treated with different Sp extracts (100 μg/mL), incubated for 24 h, and stained with Hoechst dye before imaging using an inverted fluorescence microscope. The pro-apoptotic activity of Sp extracts was observed in the following order: roots > stems > leaves. The choice of solvent significantly influenced the cytotoxic potential, with acetone-extracted fractions exhibiting the highest apoptosis induction. Notably, the water extracted from leaves did not induce apoptosis in H1975 cells.

**Figure 3:**
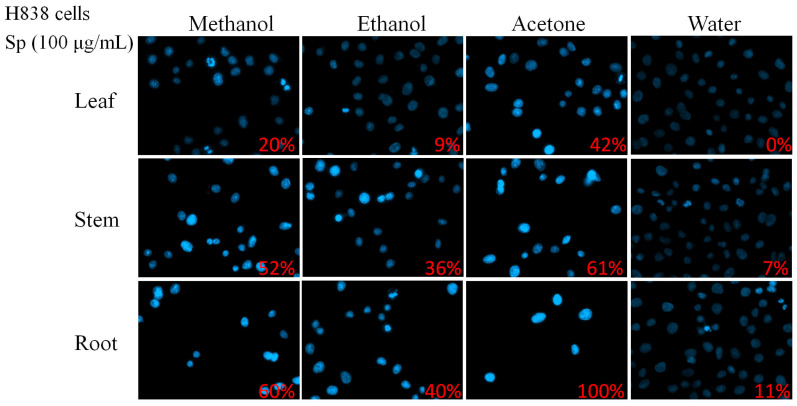
The cytotoxic effects of Sp extracts on apoptosis induction in H838 cells. The chromatin condensation assay was conducted to evaluate DNA fragmentation, which is a hallmark of apoptosis. H838 cells were treated with different Sp extracts (100 μg/mL), incubated for 24 h, and stained with Hoechst dye before imaging using an inverted fluorescence microscope. The pro-apoptotic activity of Sp extracts was observed in the following order: roots > stems > leaves and acetone > methanol > ethanol > water. The water extracted from leaves did not induce apoptosis in H838 cells.

**Figure 4:**
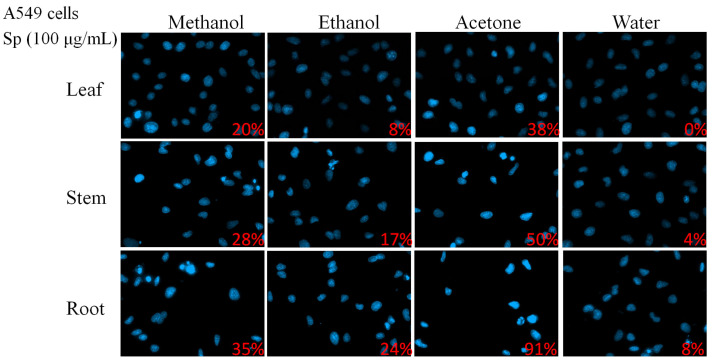
The cytotoxic effects of Sp extracts on apoptosis induction in A549 cells analyzed by Hoechst staining. A549 cells were treated with different Sp extracts (100 μg/mL) for 24 h and analyzed using Hoechst staining. Among all the extracts tested, the acetone-extracted root fraction (Sp-R-A) exhibited the most significant pro-apoptotic effects in A549 cells.

In this study, different parts of *Sarracenia purpurea* (Sp), including the leaf (Sp-L), stem (Sp-S), and root (Sp-R), were harvested, dried, finely ground, and extracted using water, methanol, ethanol, and acetone to evaluate their potential pharmacological activities (Figure 1). Prior to this investigation, little was known about the anticancer effects of Sp extracts. Given that lung cancer remains a leading cause of cancer-related mortality worldwide, with NSCLC accounting for approximately 85% of cases, we examined whether these plant extracts exhibit cytotoxic effects against NSCLC. Three NSCLC cell lines, H1975 (Figure 2), H838 (Figure 3), and A549 (Figure 4), were selected for this study.

As an initial assessment, a chromatin condensation assay using Hoechst 33342 staining was performed to determine whether the extracts induced apoptosis, a key mechanism of programmed cell death. Hoechst 33342 is a nuclear stain that selectively binds DNA with increased fluorescence intensity in apoptotic cells due to chromatin condensation. Apoptotic activity was evaluated in cells treated with extracts from different plant parts (leaf, stem, and root) and solvents (methanol, ethanol, acetone, and water) at a concentration of 100 μg/mL. Among the different extracts tested in H1975 cells, root-derived extracts exhibited the highest apoptotic induction across all solvents. Notably, the acetone-extracted root (Sp-R-A) induced complete apoptosis (100%), followed by methanol (Sp-R-M, 75%) and ethanol (Sp-R-E, 67%), whereas the water extract (Sp-R-W) demonstrated the lowest apoptotic rate (14%). A similar trend was observed in stem extracts, where acetone (Sp-S-A) induced the highest rate of apoptosis (72%), followed by methanol (Sp-S-M, 43%) and ethanol (Sp-S-E, 35%), while water (Sp-S-W) exhibited minimal apoptosis (5%). Leaf-derived extracts displayed the weakest apoptotic effects, with acetone (Sp-L-A, 48%) being the most effective, while methanol (Sp-L-M, 22%) and ethanol (Sp-L-E, 10%) exhibited moderate activity. The water extract (Sp-L-W) showed no detectable apoptotic induction (0%). These findings indicate that acetone-extracted root fractions contain the most potent bioactive compounds for apoptosis induction in H1975 cells.

To determine whether the apoptotic effects observed in H1975 cells were consistent across other NSCLC cell lines, similar experiments were conducted on H838 (Figure 3) and A549 (Figure 4) cells. Across all three NSCLC cell lines, Sp-R-A exhibited the strongest pro-apoptotic effects. Apoptosis rates reached 100% in H1975 and H838 cells, while A549 cells exhibited 91.67% apoptosis, confirming the root extract as the most potent fraction. The choice of solvent also significantly influenced the cytotoxic potential of the extracts. Acetone consistently yielded the highest apoptosis rates in all three cell lines, indicating its superior efficacy in extracting bioactive cytotoxic compounds from *S. purpurea*. Methanol and ethanol extracts also demonstrated substantial apoptotic activity, albeit at lower levels than acetone. In contrast, water extracts exhibited the weakest apoptotic effects, with no detectable apoptosis in leaf extracts and minimal effects in stem and root extracts.

The apoptotic effects of Sp extracts appeared to exhibit specificity toward NSCLC cells as variations in sensitivity were observed among the three cell lines. H1975 and H838 cells were found to be more sensitive to the extracts, displaying higher apoptotic rates across all solvent conditions. In contrast, A549 cells exhibited relatively greater resistance, particularly to methanol and ethanol extracts. Among all the extracts tested, Sp-R-A demonstrated the most significant pro-apoptotic effects, suggesting that key bioactive compounds with potent cytotoxic activity are predominantly concentrated in the roots of *S. purpurea*.

### 2.2. Cytotoxicity of Sp-R-A in NSCLC Cells Assessed by MTT Assay

**Figure 5:**
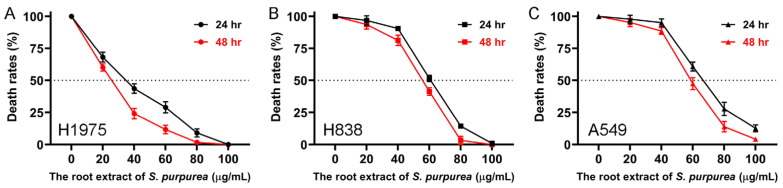
The dose-dependent cytotoxicity of Sp-R-A in NSCLC cells assessed by the MTT assay. The cytotoxic effects of Sp-R-A (0–100 μg/mL) on NSCLC cell lines were evaluated using the MTT assay at 24 and 48 h. (**A**) H1975, (**B**) H838, and (**C**) A549 cells were treated with increasing concentrations of Sp-R-A, and cell viability was assessed. The results indicate a dose-dependent decrease in cell survival, with prolonged incubation (48 h) further enhancing cytotoxicity. Among the tested cell lines, H1975 cells exhibited the highest sensitivity to Sp-R-A, with an IC_50_ value of 33.74 ± 0.82 μg/mL, followed by H838 (IC_50_ = 60.79 ± 0.54 μg/mL) and A549 (IC_50_ = 66.52 ± 0.61 μg/mL). Data represent the mean ± standard deviation from at least three independent experiments.

Due to the superior ability of Sp-R-A among the Sp extracts to induce apoptosis, its cytotoxic effects on H1975 (Figure 5A), H838 (Figure 5B), and A549 (Figure 5C) NSCLC cells were further evaluated using the MTT assay. This widely used colorimetric technique assesses cell viability by measuring mitochondrial activity as metabolically active cells convert MTT (3-[4,5-dimethylthiazol-2-yl]-2,5-diphenyl tetrazolium bromide) into insoluble formazan crystals [56]. Since mitochondrial activity is generally proportional to the number of viable cells, this assay is commonly used to assess the cytotoxic effects of potential therapeutic agents in vitro. Selected cells were treated with increasing concentrations of Sp-R-A (0–100 μg/mL) for 24 and 48 h, followed by cell viability measurements. The results demonstrated a dose-dependent reduction in cell viability, with higher extract concentrations leading to increased cytotoxicity. The half-maximal inhibitory concentration (IC_50_), representing the concentration required to reduce cell viability by 50% for 24 h, was determined for each cell line. H1975 cells exhibited the highest sensitivity to Sp-R-A, with an IC_50_ value of 33.74 ± 0.82 μg/mL, whereas H838 and A549 cells showed lower sensitivity, with IC_50_ values of 60.79 ± 0.54 μg/mL and 66.52 ± 0.61 μg/mL, respectively. Additionally, a time-dependent decrease in cell viability was observed in all cell lines, with prolonged incubation (48 h) further enhancing the cytotoxic effect. These findings indicate that Sp-R-A exerts significant cytotoxic activity against NSCLC cells, with varying degrees of sensitivity among different cell types.

### 2.3. Dose-Dependent Cytotoxicity of Sp-R-A on H838 Cell Migration and Proliferation

**Figure 6:**
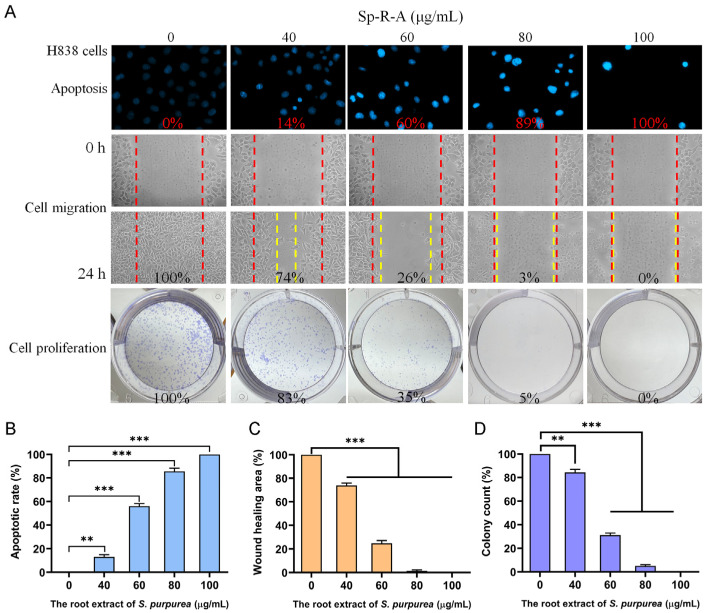
The anticancer potential of Sp-R-A in H838 cells. (**A**) The effects of Sp-R-A (0–100 μg/mL) on nuclear condensation, cell migration, and cell proliferation in H838 cells. (**B**) Hoechst staining demonstrating apoptosis and DNA fragmentation at varying concentrations of Sp-R-A. (**C**) Wound healing assay showing the migration capacity of H838 cells treated with different concentrations of Sp-R-A. Images were taken at 0 h and 24 h post-treatment. (**D**) Clonogenic assay evaluating the proliferative and colony-forming ability of H838 cells following treatment with Sp-R-A. Statistical significance is indicated as ** for *p* < 0.01 and *** for *p* < 0.001 compared to the control group.

The cytotoxic effects of Sp-R-A on apoptosis, cell migration, and proliferation in H838 cells were evaluated in a dose-dependent manner (Figure 6A). Hoechst 33342 staining revealed that treatment with Sp-R-A at concentrations of 0, 40, 60, 80, and 100 μg/mL resulted in DNA fragmentation levels of 0%, 14%, 60%, 89%, and 100%, respectively (Figure 6B), demonstrating a dose-dependent increase in apoptotic cell death. Sp-R-A also exhibited potent inhibitory effects on H838 cell migration (Figure 6C), which is a key factor in cancer metastasis. This was assessed using a wound healing assay, which is a well-established in vitro technique for evaluating cell migration in a two-dimensional setting. A uniform gap was created in a confluent monolayer of H838 cells, followed by treatment with various concentrations of Sp-R-A. The closure of this gap was monitored over 24 h, revealing a significant reduction in cell migration as the concentration of Sp-R-A increased. Specifically, Sp-R-A at 40, 60, 80, and 100 μg/mL inhibited wound closure by 26%, 74%, 97%, and 100%, respectively (Figure 6C). The observed dose-dependent suppression of migration highlights the strong anti-metastatic potential of Sp-R-A. Additionally, the effect of Sp-R-A on H838 cell proliferation was assessed using the clonogenic assay (Figure 6D), which measures the ability of single cells to survive and form colonies. The results demonstrated a substantial decrease in colony formation with increasing extract concentrations, suggesting a pronounced inhibitory effect on cell proliferative capacity. Treatment with Sp-R-A at 40, 60, 80, and 100 μg/mL led to a progressive decline in colony formation by 17%, 65%, 95%, and 100%, respectively (Figure 6D). This dose-dependent suppression of clonal expansion further supports the cytotoxic effects of Sp-R-A in H838 cells. Collectively, these findings indicate that Sp-R-A exerts significant cytotoxicity against H838 cells by inducing apoptosis, inhibiting cell migration, and suppressing proliferation. These results suggest that Sp-R-A contains bioactive compounds capable of targeting the key pathways involved in H838 cell survival and metastasis.

### 2.4. Dose-Dependent Cytotoxicity of Sp-R-A on A549 Cell Migration and Proliferation

**Figure 7:**
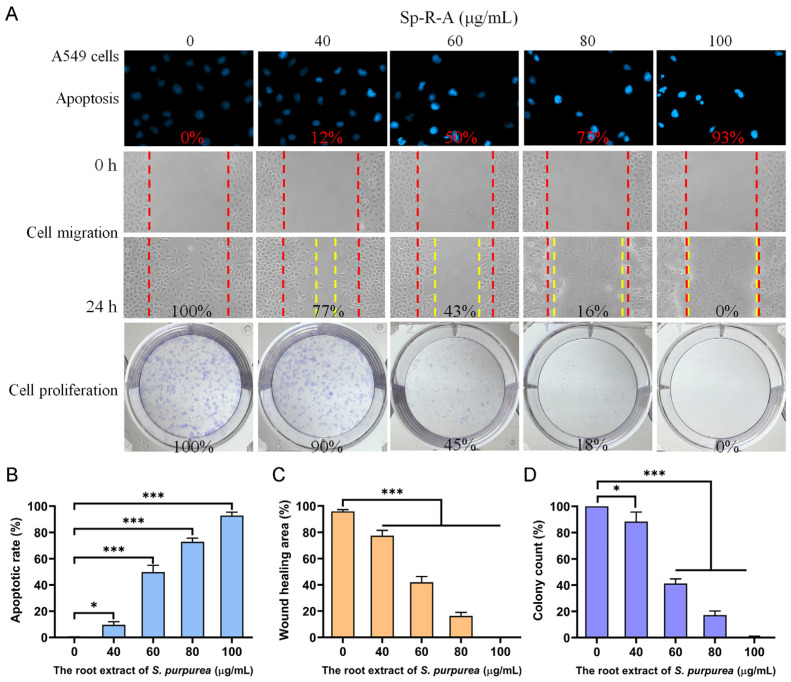
The anticancer potential of Sp-R-A in A549 cells. (**A**) The effects of Sp-R-A (0–100 μg/mL) on nuclear condensation, cell migration, and cell proliferation in A549 cells. (**B**) Hoechst staining demonstrating apoptosis and DNA fragmentation at varying concentrations of Sp-R-A. (**C**) A wound-healing assay showing the migration capacity of A549 cells treated with different concentrations of Sp-R-A. Images were taken at 0 h and 24 h post-treatment. (**D**) A clonogenic assay evaluating the proliferative and colony-forming ability of A549 cells following treatment with Sp-R-A. Statistical significance is indicated as * for *p* < 0.05 and *** for *p* < 0.001 compared to the control group.

To further investigate the cytotoxic effects of Sp-R-A, its impact on A549 cells was examined. Unlike the H838 cell line [57], which originates from the lung tissue of a 59-year-old white male diagnosed with stage 3B adenocarcinoma and exhibits epithelial morphology, A549 cells are adenocarcinomic human alveolar basal epithelial cells derived from lung tissue [58]. A comprehensive summary of the findings is presented in Figure 7A. Treatment with Sp-R-A at concentrations of 0, 40, 60, 80, and 100 μg/mL resulted in DNA fragmentation rates of 0%, 12%, 50%, 73%, and 93%, respectively, as determined by Hoechst 33342 staining (Figure 7B). In the wound healing assay, Sp-R-A significantly inhibited A549 cell migration, with wound closure rates of 100%, 77%, 43%, 16%, and 0% at the same concentrations (Figure 7C). Additionally, clonogenic assay results revealed a progressive reduction in colony formation, with inhibition rates of 0%, 10%, 55%, 82%, and 100%, respectively (Figure 7D). These findings indicate how, similar to H838 cells, A549 cells also exhibit susceptibility to the anticancer potential of Sp-R-A, highlighting its potential as a therapeutic agent against NSCLC.

### 2.5. Dose-Dependent Cytotoxicity of Sp-R-A on H1975 Cell Migration and Proliferation

**Figure 8:**
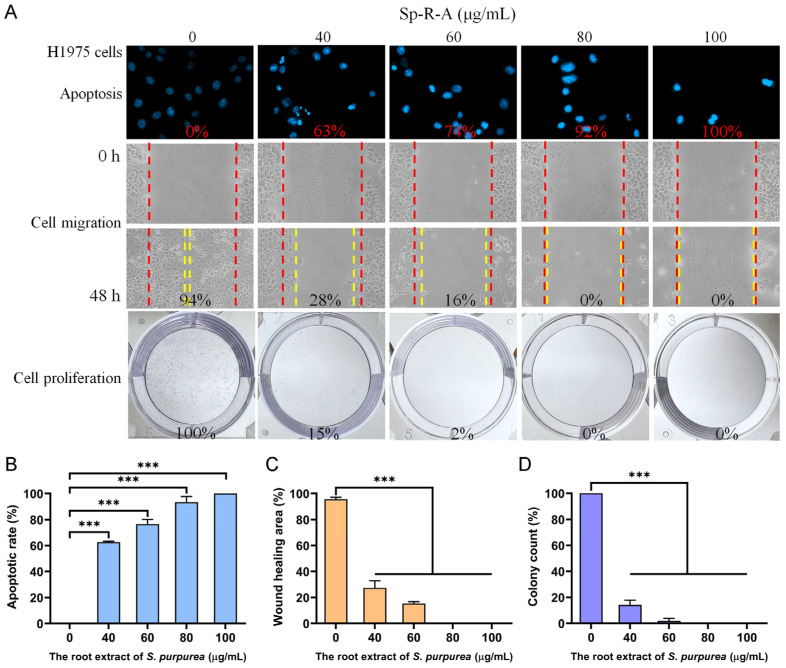
The anticancer potential of Sp-R-A in H1975 cells. (**A**) The effects of Sp-R-A (0–100 μg/mL) on nuclear condensation, cell migration, and cell proliferation in H1975 cells. (**B**) Hoechst staining demonstrating apoptosis and DNA fragmentation at varying concentrations of Sp-R-A. (**C**) A wound-healing assay showing the migration capacity of H1975 cells treated with different concentrations of Sp-R-A. Images were taken at 0 h and 48 h post-treatment. (**D**) A clonogenic assay evaluating the proliferative and colony-forming ability of H1975 cells following treatment with Sp-R-A. Statistical significance is indicated as *** for *p* < 0.001 compared to the control group.

The cytotoxic effects of Sp-R-A were further evaluated in the H1975 cell line, which originates from a non-smoking female diagnosed with NSCLC. H1975 cells [59] exhibit epithelial morphology and harbor key mutations in the epidermal growth factor receptor (EGFR) and TP53 genes, including the EGFR exon 21 L858R mutation, which is known to confer enhanced sensitivity to EGFR inhibitors, which is a common therapeutic strategy for NSCLC. A comprehensive summary of the findings is presented in Figure 8A. Treatment with Sp-R-A at concentrations of 0, 40, 60, 80, and 100 μg/mL led to DNA fragmentation rates of 0%, 63%, 74%, 92%, and 100%, respectively (Figure 8B), indicating a dose-dependent increase in apoptosis. The wound healing assay demonstrated significant inhibition of cell migration, with wound closure rates decreasing to 94%, 28%, 16%, 0%, and 0% at the corresponding extract concentrations (Figure 8C). Similarly, the clonogenic assay results showed a marked reduction in colony formation, with inhibition rates of 0%, 85%, 98%, 100%, and 100%, respectively (Figure 8D). When compared to the results obtained from H838 and A549 cells, H1975 cells exhibited the highest susceptibility to the anticancer potential of Sp-R-A, following the order H1975 > H838 > A549. These findings highlight the potential of Sp-R-A as an alternative or co-therapeutic agent for NSCLC, particularly in cases exhibiting resistance mechanisms similar to those observed in the H1975 cell line.

### 2.6. The Effect of Extracting Solvents on the Apoptosis-Inducing Potential of Sp-R Extracts in H1975 Cells

**Figure 9:**
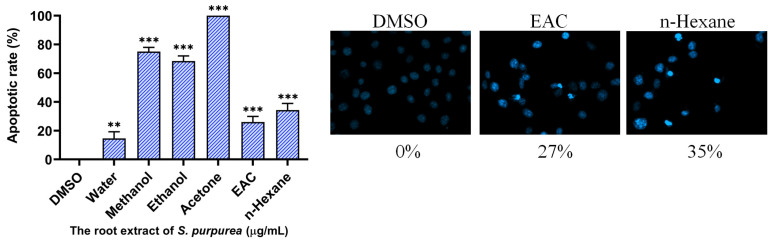
The influence of solvent selection on the apoptosis-inducing potential of Sp-R extracts in H1975 cells. Hoechst staining was performed to evaluate nuclear condensation in H1975 cells treated with Sp-R extracts prepared using different solvents, including DMSO, distilled water, methanol, ethanol, acetone, EAC, and n-hexane. Among these, the acetone extract exhibited the highest apoptotic effect. Statistical significance is indicated as ** for *p* < 0.01 and *** for *p* < 0.001 compared to the control group.

Sp-R-A, extracted using the polar aprotic solvent acetone, was identified as the most potent extract with anticancer potential (Figure 2). To further investigate the influence of solvent choice, the apoptosis-inducing effects of other solvents, including the polar aprotic solvent ethyl acetate (EAC) and the non-polar solvent n-hexane, were also tested on H1975 cells (Figure 9). At a concentration of 100 μg/mL, extracts prepared using DMSO, distilled water, methanol, ethanol, acetone, EAC, and n-hexane induced apoptosis in H1975 cells at rates of 0%, 14%, 75%, 67%, 100%, 27%, and 35%, respectively. These results provided the relative effectiveness of Sp-R extracts in the following order: acetone > methanol > ethanol > n-hexane > EAC > distilled water > DMSO. Accordingly, the choice of solvent is a critical factor influencing the bioactivity of the extract, although its effectiveness does not appear to be strictly correlated with the chemical classification of the solvent used.

### 2.7. The Synergistic Apoptosis-Inducing Potential of the Combination of Afatinib and Sp-R-A in H1975 Cells

**Figure 10:**
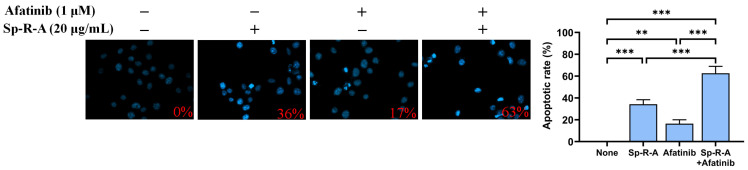
The synergistic effect of Sp-R-A and afatinib on apoptosis in H1975 cells. The combined effect of Sp-R-A (20 μg/mL) and afatinib (1 μM) on apoptosis in H1975 cells was evaluated using Hoechst staining. The results indicate that co-treatment with afatinib and Sp-R-A significantly enhances apoptosis, suggesting a potential synergistic therapeutic strategy against lung cancer. Statistical significance is indicated as ** for *p* < 0.01 and *** for *p* < 0.001 compared to the control group.

We further investigated whether the combination of Sp-R-A and the chemotherapeutic agent afatinib exerts a synergistic effect on apoptosis in H1975 cells (Figure 10). Afatinib [60], marketed as Gilotrif, is an epidermal growth factor receptor (EGFR) tyrosine kinase inhibitor administered orally for the treatment of NSCLC cases with specific EGFR mutations. Our findings demonstrated an enhanced therapeutic effect when afatinib was combined with Sp-R-A in H1975 cells. Treatment with 20 μg/mL Sp-R-A alone, which exhibited minimal cytotoxicity, resulted in 36% apoptosis, while 1 μM afatinib alone induced 17% apoptosis. However, co-treatment with both Sp-R-A and afatinib significantly increased apoptosis to 63%, indicating a synergistic cytotoxic effect. The combination of treatments enhanced the apoptotic rate from 17% (afatinib alone) to 63% when combined with 20 μg/mL Sp-R-A, suggesting its potential as a co-therapeutic strategy for NSCLC.

### 2.8. Inhibition of Human RPA32 by sp Acetone Extracts

**Figure 11:**
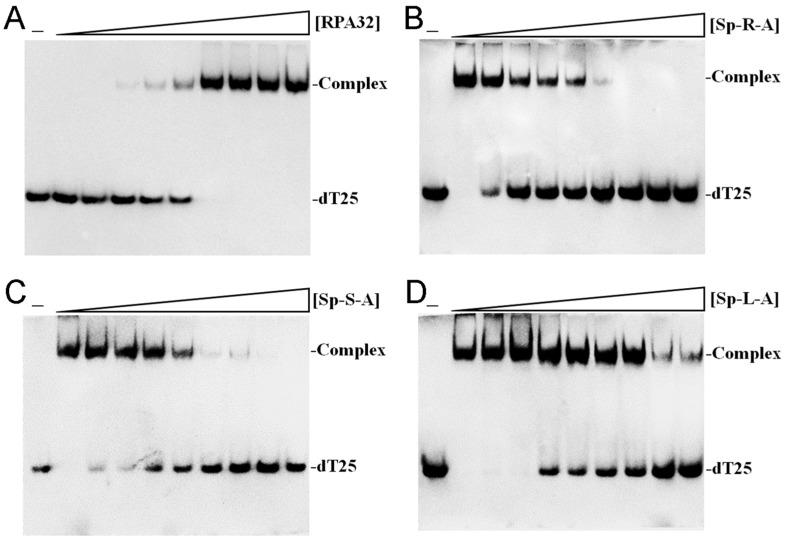
The inhibition of huRPA32 DNA-binding activity by Sp acetone extracts. (**A**) The binding of huRPA32 to ssDNA dT25. Purified huRPA32 (0, 0.31, 0.63, 1.25, 2.5, 5, 10, 20, 40, and 50 μM) was incubated with biotin-labeled ssDNA dT25, and its binding was analyzed using EMSA. The binding constant ([Protein]_50_) of huRPA32 was quantified through linear interpolation based on protein concentrations. (**B**–**D**) The inhibition of huRPA32 DNA-binding activity by *S. purpurea* extracts from different plant parts: (**B**) root extract (Sp-R-A), (**C**) stem extract (Sp-S-A), and (**D**) leaf extract (Sp-L-A). These were all prepared using acetone as the extraction solvent. huRPA32 (10 μM) was incubated with ssDNA dT25 in the presence of increasing extract concentrations (0, 7.5, 15.1, 31.3, 62.5, 125, 250, 500, and 1000 μg/mL). A final concentration of 1% DMSO was used as a negative control (corresponding to 0 μg/mL extract). The leftmost lane (“−”) denotes the absence of both huRPA32 and extract in the incubation reaction with dT25.

The Sp extract exhibited significant cytotoxic effects on lung cancer cells, including apoptosis induction and the inhibition of cell proliferation. These findings suggest that the extract may contain bioactive compounds capable of disrupting key cellular growth and replication pathways. Therefore, we investigated whether human RPA32 (huRPA32), a crucial protein in DNA replication due to its role in binding ssDNA, is a target for inhibition by different Sp extracts obtained using acetone. The electrophoretic mobility shift assay (EMSA) was used to evaluate the binding activity of recombinant huRPA32 (0–50 μM) to ssDNA, specifically the deoxythymidine homopolymer dT25. huRPA32 was functionally expressed and purified from *Escherichia coli* [61]. EMSA relies on the principle that the stable protein–DNA complex formation results in reduced electrophoretic mobility compared to unbound DNA, indicating successful binding. The incubation of huRPA32 with dT25 led to a distinct band shift, confirming its binding activity (Figure 11A). The EMSA titration curve determined the midpoint value for ssDNA binding ([Protein]_50_) of huRPA32 to dT25 as 5.7 ± 0.6 μM (Figure 11A). A concentration of 10 μM huRPA32, which resulted in complete binding to dT25, was selected for further inhibition studies using Sp extracts (0–1000 μg/mL). The titration curves for Sp-R-A (Figure 11B), Sp-S-A (Figure 11C), and Sp-L-A (Figure 11D) were analyzed to determine the IC_50_ values for huRPA32 ssDNA-binding inhibition. The IC_50_ values were 13.6 ± 1.6 μg/mL for Sp-R-A, 43.0 ± 2.8 μg/mL for Sp-S-A, and 236 ± 34 μg/mL for Sp-L-A. These results suggest that specific bioactive compounds within the root extract of *S. purpurea*, particularly those extracted using 100% acetone, may contribute individually or synergistically to its potent inhibitory activity against huRPA32.

### 2.9. AlphaFold Prediction of huRPA32 Structure and Molecular Docking of Potential Inhibitors

**Figure 12:**
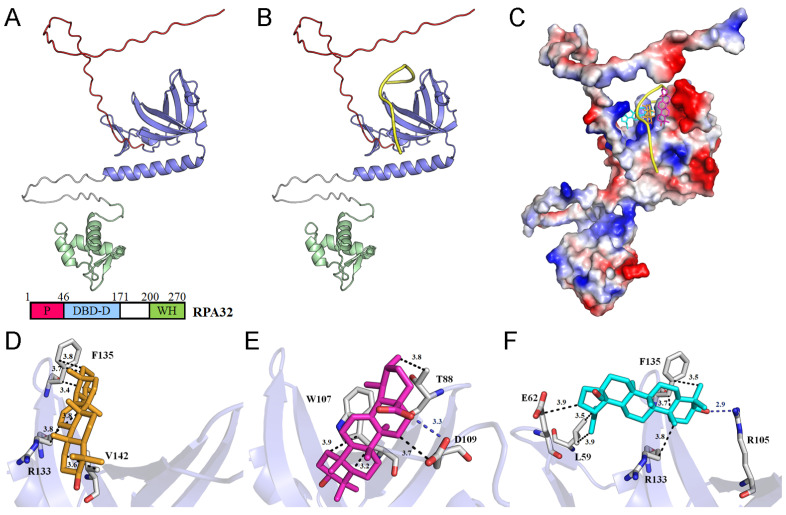
AlphaFold prediction of huRPA32 structure and molecular docking of potential inhibitors. (**A**) AlphaFold 3.0 prediction of the full-length huRPA32 structure based on its amino acid sequence. The protein domains are color-coded: the P domain is shown in red; the DBD-D domain is shown in blue; and the WH domain is shown in green. The ssDNA-binding site is known to be located within the DBD-D domain. (**B**) The AlphaFold prediction of huRPA32 in complex with ssDNA dT10 and ssDNA depicted in yellow. (**C**) The docking analysis of the three compounds with the highest binding affinity, as determined by AutoDock Vina: α-amyrin (light orange), ursolic acid (light magenta), and betulinaldehyde (cyan). These compounds target different ssDNA-binding sites in huRPA32, potentially hindering its ability to bind ssDNA. The charge distribution pattern is shown to highlight the predicted ssDNA-binding sites. (**D**) The binding mode of α-amyrin. α-Amyrin is positioned within the ssDNA-binding cavity and interacts hydrophobically with Arg133, Phe135, and Val142 of huRPA32. The numbers within the black dashed lines indicate the interaction distances. (**E**) The binding mode of ursolic acid. Ursolic acid forms a hydrogen bond with Asp109 and engages in hydrophobic interactions with Thr88, Trp107, and Asp109. (**F**) Binding mode of betulinaldehyde. Betulinaldehyde establishes a hydrogen bond with Arg105 and interacts hydrophobically with Leu59, Glu62, Arg133, and Phe135.

Previous mass spectrometry analyses from different laboratories have identified several major compounds in *S. purpurea*, including betulinaldehyde, β-sitosterol, betulinic acid, ursolic acid, quercetin-3-O-galactoside, morroniside, stigmast-5-en-3-ol, 7,8-dihydro-α-ionone, α-amyrin, and botulin [8,11,55]. These compounds may interact with and inhibit huRPA32 either individually or synergistically. To further investigate these potential interactions, in silico molecular docking experiments were conducted (Table 1). Since the full-length structure of huRPA32 remains unavailable, we used AlphaFold to generate a predicted structure in this study. AlphaFold’s AI-driven predictions are now widely recognized for their high accuracy, as demonstrated by its 2024 Nobel Prize in Chemistry recognition [62]. The predicted molecular structure of full-length huRPA32 (Figure 12A) and its complex with ssDNA dT10 (Figure 12B) was generated using AlphaFold 3.0 [63,64], enabling molecular docking studies to predict binding sites and calculate binding affinities using AutoDock Vina. The predicted DNA-binding site of huRPA32 was characterized by a positively charged surface, which was occupied by ssDNA in the model (Figure 12C). Based on docking analysis, the binding affinities of these compounds to huRPA32 are presented in the following ranking: α-amyrin > ursolic acid > betulinaldehyde > botulin > betulinic acid > stigmast-5-en-3-ol > β-sitosterol > quercetin-3-O-galactoside > morroniside > 7,8-dihydro-α-ionone (Table 1). Notably, α-amyrin, ursolic acid, and betulinaldehyde exhibited the highest binding affinities and were further analyzed for their binding interactions. Alpha-amyrin was positioned within the ssDNA-binding cavity and interacted hydrophobically with Arg133, Phe135, and Val142 of huRPA32 (Figure 12D). Ursolic acid formed a hydrogen bond with Asp109 and engaged in hydrophobic interactions with Thr88, Trp107, and Asp109 (Figure 12E). Betulinaldehyde established a hydrogen bond with Arg105 and formed hydrophobic interactions with Leu59, Glu62, Arg133, and Phe135 (Figure 12F). Given these diverse interactions at distinct ssDNA-binding sites, the inhibitory potential of huRPA32 may result from the combined effects of these compounds. However, further biochemical and structural studies are required to validate these findings and confirm their inhibitory mechanisms.

## 3. Discussion

Cancer is characterized by uncontrolled cell proliferation and the ability of malignant tumors to spread locally or systemically, making it one of the leading causes of mortality worldwide, with lung cancer posing the greatest threat [60,65,66,67,68,69,70,71]. Natural compounds and plant extracts are increasingly being explored as potential therapeutic agents against lung cancer, offering alternative treatment strategies and addressing challenges such as drug resistance, which remains a major hurdle in NSCLC treatment [72,73,74,75,76,77,78,79]. In this study, we systematically screened different plant parts of the carnivorous plant *S. purpurea* (leaf, stem, and root) using various extraction solvents (Figure 1) to assess their cytotoxic effects on NSCLC cell lines: H1975 (Figure 2), H838 (Figure 3), and A549 (Figure 4). Our findings demonstrate that beyond the well-known digestive function of the pitcher-shaped leaves—such as protease-mediated prey digestion [14,80,81] for nutrient acquisition—the plant’s body, particularly its roots, exhibits significant cytotoxic activity (Figure 5). The root extracts effectively suppressed cancer cell proliferation and strongly induced apoptosis (Figure 6, Figure 7, Figure 8 and Figure 9). Furthermore, we identified huRPA32 as a potential molecular target of *S. purpurea* extracts, confirmed through EMSA (Figure 11) and in silico molecular docking analysis (Table 1 and Figure 12). Notably, the apoptosis-inducing effect of Sp-R-A was significantly enhanced when combined with the clinically approved EGFR-targeting lung cancer drug afatinib, suggesting a potential synergistic effect (Figure 10). These findings highlight the promising role of the *S. purpurea* root extract as a potential co-therapeutic agent for NSCLC and warrant further investigation into its mechanism of action and clinical applicability.

Since Charles Darwin’s seminal work on carnivorous plants has sparked interest and debate regarding their evolutionary adaptations, these unique plants have continued to fascinate researchers [81,82]. More recently, there has been growing interest in their potential as a source of secondary metabolites with significant biological activity, particularly in cancer research [5,61,82,83,84,85,86,87]. Various bioactive compounds, including flavonoids, naphthoquinones, alkaloids, and terpenoids, have been identified in carnivorous plants, demonstrating notable cytotoxic effects against cancer cells [5,82,83]. For example, naphthoquinones such as plumbagin, droserone, and ramentaceone—found in *Drosera* species and *Nepenthes*—exhibit strong cytotoxicity by inducing reactive oxygen species (ROS) generation, DNA damage, and apoptosis in cancer cells. Flavonoids, including quercetin, rutin, kaempferol, and luteolin, identified in *Nepenthes* and *Sarracenia*, possess antioxidant, anti-inflammatory, and apoptosis-inducing properties that contribute to the inhibition of cancer cell proliferation. Triterpenoids, such as ursolic acid, betulinic acid, α-amyrin, and β-amyrin, detected in *S. purpurea*, are known for their ability to inhibit cancer cell migration and proliferation while promoting apoptosis through mitochondrial pathways. Mass spectrometry analyses from different studies have identified several major bioactive compounds in *S. purpurea*, including betulinaldehyde, β-sitosterol, betulinic acid, ursolic acid, quercetin-3-O-galactoside, morroniside, stigmast-5-en-3-ol, 7,8-dihydro-α-ionone, α-amyrin, and botulin [8,11,55]. Accordingly, some of these compounds in the extract of *S. purpurea* likely contribute individually or synergistically to the antiproliferative and apoptosis-inducing effects observed in this study (Figure 6, Figure 7 and Figure 8). A key advantage of using natural plant extracts for cancer treatment is their multi-targeted mode of action, which may help overcome drug resistance by simultaneously disrupting multiple signaling pathways [23,24,25]. Given these properties, certain bioactive compounds from *S. purpurea* could be considered in combination with conventional chemotherapy to enhance therapeutic efficacy against cancer.

Afatinib [60], marketed as Gilotrif and classified as an anilinequinazoline derivative, is a member of the tyrosine kinase inhibitor family and is commonly administered orally for the treatment of NSCLC cases with EGFR mutations. Previous studies have demonstrated afatinib’s synergistic potential when combined with various agents, including romidepsin [88], MRTX1133 [89], aspirin [90], vinorelbine [91], and SU11274 [92], in lung cancer therapy. In this study, we observed a synergistic effect between Sp-R-A and afatinib in enhancing apoptosis in H1975 cells (Figure 10). Given that H1975 cells harbor the EGFR L858R and T790M mutations [59], which confer resistance to first-generation EGFR inhibitors, the ability of Sp-R-A to enhance afatinib-induced apoptosis is of significant therapeutic relevance. This finding supports the potential of Sp-R-A as a complementary agent in NSCLC therapy, particularly in cases exhibiting resistance to conventional targeted treatments. Although it remains unclear whether Sp-R-A directly inhibits EGFR tyrosine kinase, the observed synergistic interaction suggests that Sp-R-A and its bioactive compounds may exert anticancer effects through alternative mechanisms. Possibly, while afatinib inhibits EGFR tyrosine kinase, Sp-R-A may target other essential cellular processes, such as DNA replication and repair, mediated by RPA, as demonstrated in Figure 11. Consequently, these two agents may simultaneously target distinct critical pathways, contributing to their significant combined effect. However, further experimental and clinical studies are necessary to validate these synergistic effects.

Previous studies have demonstrated that *S. purpurea* extract can inhibit the ssDNA-binding activity of SSB from *Staphylococcus aureus* [37]. This finding led us to investigate whether the extract exhibits cytotoxic effects on human NSCLC cells through the suppression of the eukaryotic SSB, namely RPA [93,94]. Defects in the DNA damage response contribute to genomic instability, which is a hallmark of tumor development [95]; however, these same vulnerabilities can be exploited for cancer therapy [47,51,52,96]. One of the most compelling findings of this study is the inhibition of huRPA32, which is a crucial ssDNA-binding protein involved in DNA replication, repair, and genome maintenance [49,97,98,99]. The identification of huRPA32 as a molecular target of Sp-R-A aligns with emerging research highlighting the role of replication stress in cancer therapy (Figure 11). The selective inhibition of huRPA32 is particularly significant, as normal human cells do not require continuous DNA synthesis, whereas rapidly proliferating cancer cells depend on sustained genome replication. Thus, targeting huRPA32 could preferentially induce cytotoxicity in cancer cells while sparing normal tissues. In this study, the molecular mechanism underlying Sp-R-A’s cytotoxicity was investigated through its inhibitory activity against huRPA32 using EMSA. The results demonstrated that Sp-R-A effectively blocked the ssDNA-binding function of huRPA32 with an IC_50_ of 13.6 ± 1.6 μg/mL, which was significantly lower than the IC_50_ values observed for extracts from other plant parts, such as 43.0 ± 2.8 μg/mL for Sp-S-A and 236 ± 34 μg/mL for Sp-L-A (Figure 11). Given the essential role of RPA32 in DNA replication, repair, and genomic stability, its inhibition can induce replication stress, impair tumor cell survival, and enhance the efficacy of DNA-damaging agents. AlphaFold structural prediction and molecular docking studies further supported the hypothesis that key bioactive compounds in *S. purpurea*, such as α-amyrin, ursolic acid, and betulinaldehyde, strongly interact with the DNA-binding domain of huRPA32, potentially contributing to its inhibition (Table 1 and Figure 12). Although the full-length structure of huRPA32 is not available in the PDB, AlphaFold’s AI-driven predictions are now widely recognized for their high accuracy, even for targets without experimentally determined structures, as evidenced by its recognition in the 2024 Nobel Prize in Chemistry [62,63,64,100,101]. The identification of these molecular interactions provides valuable insight into the structure–function relationship of Sp-R-A compounds and their potential as huRPA32 inhibitors. These findings suggest that *S. purpurea* may serve as a promising source of novel anticancer agents targeting DNA replication machinery in NSCLC therapy and potentially other cancer treatments.

Our findings indicate that Sp-R-A exhibits potent cytotoxic activity in NSCLC cells by inducing apoptosis, inhibiting cell proliferation, and suppressing cell migration. The cytotoxic effects were dose-dependent and varied among cell lines, with H1975 cells displaying the highest sensitivity (IC_50_ = 33.74 ± 0.82 μg/mL), followed by H838 (IC_50_ = 60.79 ± 0.54 μg/mL) and A549 (IC_50_ = 66.52 ± 0.61 μg/mL). Chromatin condensation assays confirmed significant nuclear fragmentation in treated cells, which is a hallmark of apoptosis. However, Sp-R-A did not induce a similar apoptotic effect in normal 16HBE cells at a concentration of 40 μg/mL (Figure S1). Additionally, migration and clonogenic assays revealed a pronounced inhibitory effect of Sp-R-A on tumor cell motility and colony formation, suggesting its potential to impede NSCLC metastasis and tumor progression. While the root extract (Sp-R) exhibited the strongest cytotoxic effects, extracts from other plant parts, such as the stem and leaf, demonstrated comparatively lower activity. Moreover, the choice of extraction solvents significantly influenced the bioactivity of Sp-R. Acetone-extracted root fractions exhibited the highest cytotoxic effects, whereas water extracts showed minimal activity. The relative effectiveness of Sp-R extracts was found in the following order: acetone > methanol > ethanol > n-hexane > EAC > distilled water > DMSO (Figure 9). This underscores the importance of solvent selection in optimizing the yield of bioactive compounds and enhancing the anticancer potential of Sp-R. Furthermore, although acetone is less safe than ethanol as an extraction solvent, future applications of Sp-R-A should ensure the complete removal of residual acetone to maintain safety for therapeutic use.

While this study investigated the inhibitory potential of Sp-R-A on huRPA32, recent research has demonstrated that extracts from the carnivorous plant *Nepenthes miranda* can also inhibit the ssDNA-binding activity of huRPA32 [61]. The reported IC_50_ values for *N. miranda* extracts were 706.6 ± 32.6 μg/mL for the pitcher, 231.4 ± 12.5 μg/mL for the leaf, and 101.6 ± 6.3 μg/mL for the stem, with the stem extract exhibiting the most significant inhibitory effect [61]. An analysis of the chemical composition between these extracts revealed two overlapping compounds: stigmast-5-en-3-ol and α-amyrin. Notably, molecular docking using AutoDock predicted α-amyrin to have the highest binding affinity to huRPA32 (−9.0 kcal/mol) among the tested compounds (Table 1). While this suggests that α-amyrin may contribute to the inhibition of huRPA32’s ssDNA-binding activity, the IC_50_ value of Sp-R-A (13.6 ± 1.6 μg/mL) is more than seven times lower than that of the *N. miranda* stem extract, indicating a significantly stronger inhibitory effect. Consequently, Sp-R-A appears to be a more promising candidate than the *N. miranda* stem extract for identifying potent huRPA32 inhibitors. Further research should first focus on experimentally validating the inhibitory potential of α-amyrin against huRPA32. Our lab recently purchased the compounds identified in Sp-R-A for the detailed analysis of their inhibitory effects on huRPA32.

Overall, our study provides novel evidence supporting the cytotoxic potential of the *S. purpurea* root extract against NSCLC and identifies huRPA32 as a promising molecular target. The ability of Sp-R-A to induce apoptosis, inhibit migration, suppress proliferation, and enhance the efficacy of afatinib suggests its potential therapeutic value. Further studies are warranted to isolate and characterize the active compounds responsible for huRPA32 inhibition and to assess their pharmacokinetics, bioavailability, and in vivo efficacy. Given the increasing interest in natural product-based anticancer strategies, Sp-R-A represents a promising candidate for future drug development and combination therapies targeting NSCLC.

## 4. Materials and Methods

### 4.1. Materials and Cell Culture

All solvents and chemicals were of the highest grade and commercially obtained from Sigma-Aldrich (St. Louis, MO, USA). The cell lines used in this study were kindly provided by Dr. Kuo-Ting Chang (Taoyuan General Hospital). The H1975, A549, and H838 cells were maintained in RPMI 1640 supplemented with 10% FBS, 100 units/mL penicillin, and 100 μg/mL streptomycin. Cells were incubated at 37 °C in a 95% air and 5% CO_2_ atmosphere.

### 4.2. Expression and Purification of the Recombinant Protein

The plasmid pET21e-huRPA32 [61] was used to transform *E. coli* BL21 (DE3) cells, which were cultured in an LB medium supplemented with 250 μg/mL ampicillin and incubated at 37 °C under vigorous shaking until an OD_600_ of 0.9 was reached. Recombinant huRPA32 expression was induced with 1 mM IPTG. After induction, cells were harvested, resuspended in a sonication buffer (20 mM Tris–HCl, 0.5 M NaCl, pH 8.0), and lysed by sonication. The pellet was collected and washed with a sonication buffer containing 2 M urea before being harvested again. The resulting pellet was solubilized in a sonication buffer supplemented with 8 M urea and incubated for 12 h at 4 °C. The solution was then dialyzed twice daily against a refolding buffer (5 mM imidazole, 20 mM Tris–HCl, 0.5 M NaCl, and 1 mM DTT, pH 7.4). Subsequently, the protein solution was dialyzed against a purification buffer (5 mM imidazole, 20 mM Tris–HCl, and 0.5 M NaCl, pH 7.9) for 4 h. Recombinant huRPA32 was purified from the soluble supernatant using Ni^2+^-affinity chromatography (HisTrap HP; GE Healthcare Bio-Sciences, Piscataway, NJ, USA), and eluted with an elution buffer containing 20 mM Tris-HCl, 250 mM imidazole, and 0.5 M NaCl (pH 7.9). The purified protein was dialyzed against a storage buffer (40 mM Tris-HCl, 50 mM NaCl, pH 7.5) and concentrated to 15 mg/mL for future use. Protein purity was confirmed via electrophoresis on 12% SDS-PAGE gel.

### 4.3. Biorad Protein (Bradford) Assay

The dye reagent was prepared by diluting one part of the Dye Reagent Concentrate with four parts of deionized water, followed by filtration through a 0.25 μm filter to eliminate any particulates. A bovine serum albumin protein standard was prepared at concentrations ranging from 0.1 to 0.5 mg/mL and analyzed in duplicate. For each standard and sample solution, 10 μL was transferred into individual wells of a microtiter plate, followed by the addition of 200 μL of the diluted dye reagent. The solutions were thoroughly mixed using a multichannel pipette. The plate was then incubated at room temperature for a minimum of 5 min before absorbance was measured at 595 nm.

### 4.4. EMSA

A biotinylated dT25 was used as a standard assay substrate to analyze huRPA32 binding via the LightShift Chemiluminescent EMSA Kit [102]. Purified huRPA32 was incubated with ssDNA (30 fmol/μL) at varying concentrations (0, 0.31, 0.63, 1.25, 2.5, 5, 10, 20, 40, and 50 μM). The huRPA32–dT25 mixtures were incubated for 60 min at 37 °C in a buffer containing 40 mM Tris–HCl (pH 7.5) and 50 mM NaCl. After the addition of the dye mixture, the reaction samples were resolved on 8% native polyacrylamide gel. Electrophoresis was performed at 100 V for 1 h in a TBE running buffer. The protein–DNA complexes were subsequently transferred onto a positively charged nylon membrane (GE, Boston, MA, USA) via electroblotting. The cross-linking of the transferred DNA to the membrane was carried out using a UV-light instrument equipped with 312 nm bulbs, with an exposure time of 10 min. DNA detection was performed using a streptavidin–horseradish peroxidase conjugate and a chemiluminescent substrate (Pierce Biotechnology, Waltham, MA, USA).

### 4.5. huRPA32 Inhibition

An inhibition assay was performed by incubating huRPA32 (10 μM) with both dT25 and varying concentrations of the *S. purpurea* extract (0, 7.5, 15.1, 31.3, 62.5, 125, 250, 500, and 1000 μg/mL). Following EMSA, a titration curve was generated based on experimental data. The IC_50_ value of *S. purpurea* for huRPA32 inhibition was directly determined through graphical analysis.

### 4.6. Plant Materials and Extract Preparations

The plant material was obtained from Guoguang Flower Market and Taiwan Provincial Flower Marketing Cooperative and was identified by Dr. Zhong-Bao Zhang in November 2022. The leaves, stems, and roots of *S. purpurea* were collected, dried, cut into small pieces, and ground into a fine powder (Figure 1). One gram of plant powder was placed into a 250 mL conical flask, to which 100 mL of the designated 100% solvent was added. The flask was then shaken on an orbital shaker for 5 h. The resulting extract was filtered through a 0.45 μm filter, and the solvent was subsequently evaporated using a hot air circulation oven set at 40 °C. The extracts were stored at −80 °C until use. To prepare a stock solution, the powder extract was dissolved in 20% DMSO at a final concentration of 20 mg/mL. For anticancer cell assays, the stock solution was diluted with a supplemented culture medium to achieve the desired assay concentrations. Cancer cells were then incubated with these extract solutions, while the culture medium containing 0.2% DMSO served as the control group. For EMSA, the stock solution was diluted with a reaction buffer (40 mM Tris-HCl, 50 mM NaCl, pH 7.5) to the designated assay concentrations. The protein was then incubated with these extract solutions, while the reaction buffer containing 1% DMSO served as the control group.

### 4.7. MTT Cell Viability Assay

The effect of the *S. purpurea* extract on the viability of the selected cells was evaluated using the MTT assay [103]. Serial dilutions of the extract were prepared and added to the cell culture medium in a 96-well plate, with each well containing the selected cells in a final volume of 100 μL. After 48 h of incubation, 30 μL of the MTT solution (5 mg/mL MTT in PBS) was added to each well. The treated cells were then incubated with the MTT solution for an additional 4 h at 37 °C in the dark. The resulting formazan crystals were dissolved in 100 μL of DMSO at 37 °C for 10 min. Absorbance was measured at 540 nm using a plate spectrophotometer. All assays were performed in triplicate.

### 4.8. Chromatin Condensation Assay

Apoptosis in cancer cells was assessed using the Hoechst 33342 staining method [104]. The selected cells were seeded in 96-well plates at a density of 5 × 10^3^ cells per well and allowed to adhere for 16 h before being incubated with the extract for 24 h. Following incubation, the cells were washed with PBS and stained with Hoechst dye (1 μg/mL) in the dark for 10 min. Stained cells were then imaged using the ImageXpress Pico system (Molecular Devices, San Jose, CA, USA) equipped with DAPI filter cubes.

### 4.9. Clonogenic Formation Assay

The inhibition of cell proliferation was evaluated using a clonogenic formation assay [105]. The selected cells were seeded at a density of 1 × 10^3^ cells per well in 6-well plates and incubated overnight to allow for attachment. Following treatment with the extract for 5–7 days, the cells were washed with PBS, fixed with methanol, and stained with 0.5% crystal violet for 20 min. Colony formation was assessed by counting the number of colonies under a light microscope to determine the extent of proliferation inhibition.

### 4.10. Wound-Healing Assay

The inhibition of cell migration was evaluated using a wound-healing assay, which is a technique commonly used to assess collective cell migration in a two-dimensional environment [106]. A cell-free gap was introduced in a confluent monolayer of cells, stimulating migration to close the wound. The selected cells were cultured in a serum-reduced medium for 6 h before creating a linear wound using a pipette tip. After washing with a serum-reduced medium, the cells were treated with the extract for 24 h, and their migration ability was subsequently assessed.

### 4.11. Binding Analysis Using AutoDock Vina

The interaction between compounds and huRPA32 was analyzed using AutoDock Vina (v1.2.2) [107,108,109]. The structure of huRPA32 was predicted using AlphaFold 3 [63] by inputting its full-length amino acid sequence, with dT10 serving as the bound ssDNA. Pre-docking preparations, including charge assignments and volume measurements, were conducted using AutoDockTools (v1.5.6). The 2D structures of the compounds were obtained from PubChem and converted into the sdf format. Both the ligand molecules and the protein target were then prepared as PDBQT files for docking using AutoDock Vina through the PyRx Virtual Screening Tool (v1.1). The docking results were visualized using PyMOL v2.2.0 software.

### 4.12. Statistical Analysis

Experiments were conducted in triplicate, and the results are presented as the mean ± standard deviation (SD). Statistical significance (*p* < 0.05) was assessed using GraphPad Prism 5 (GraphPad Software Inc., San Diego, CA, USA). One-way ANOVA was employed to evaluate the differences between means.

## 5. Conclusions

The increasing interest in carnivorous plant extracts for biomedical applications is driven by their ability to produce unique secondary metabolites with potent biological activities. We found that the *S. purpurea* root extract exhibits significant anticancer potential by inducing apoptosis, suppressing cell migration and proliferation, and inhibiting huRPA32, making it a promising candidate for further research in oncology and natural product-based drug development. Additionally, the apoptosis-inducing effect of the root extract was significantly enhanced when combined with afatinib. Future studies should focus on isolating specific active compounds, optimizing their efficacy, and evaluating their therapeutic potential in vivo.

## Figures and Tables

**Table 1:** Molecular docking analysis of huRPA32.

Compound	Affinity (kcal/mol)
Betulinaldehyde	−8.1
β-Sitosterol	−7.0
Betulinic acid	−7.4
Ursolic acid	−8.8
Quercetin-3-O-galactoside	−6.7
Morroniside	−5.9
Stigmast-5-en-3-ol	−7.2
7,8-Dihydro-alpha-ionone	−4.5
α-Amyrin	−9.0
Betulin	−7.9

## Data Availability

The data are contained within the article.

## Supplementary Materials

The following supporting information can be downloaded at https://www.mdpi.com/article/10.3390/plants14101426/s1. Figure S1: Effects of Sp-R-A treatment on normal 16HBE cells.
