# Supplementary material for: Inhibition of RPA32 and Cytotoxic Effects of the Carnivorous Plant Sarracenia purpurea Root Extract in Non-Small-Cell Lung Cancer Cells
Source: Plants (Basel). 2025 May 9;14(10):1426. doi: 10.3390/plants14101426 (PMC12115182; doi:10.3390/plants14101426)
Supplement: Supplementary file 1 [file plants-14-01426-s001.zip › plants-3572349-supplementary.pdf]

Figure S1. Effects of Sp-R-A treatment on normal 16HBE cells.

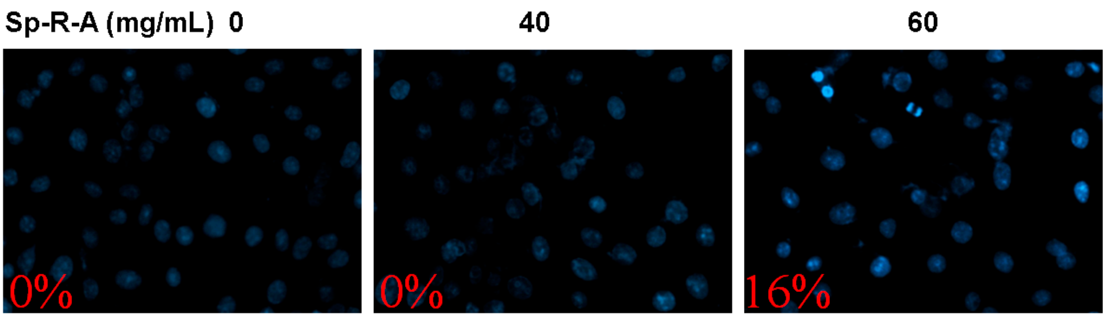

Figure S1. Effects of Sp-R-A treatment on normal 16HBE cells. Sp-R-A was applied at varying concentrations. At 40 μg/mL, no significant apoptosis was observed, whereas treatment at 60 μg/mL induced approximately 16% apoptosis in 16HBE cells.
